# "A Children's Hospital in Difficulties"

**Published:** 1917-10-06

**Authors:** E. D. Spence

**Affiliations:** Baildon


### THE EDITOR'S LETTER-BOX.

### "A Children's Hospital in Difficulties."

*To the Editor of* The Hospital.

Sir,—I have read your comments in your issue of August 11 and September 15 *re* the remarks I made at the Bradford Hospital Fund quarterly meeting on July 26, and I think your remarks call for a personal explanation on my part, as evidently you do not understand my position on the Bradford Children's Hospital Board.

I am elected by the Bradford Hospital Fund as one of their representatives on the Children's Hospital Board, and, as such, report any matters of interest to the Bradford Hospital Fund (which, by the way, is mostly composed of representatives of the workpeople of the city of Bradford). I was speaking to them when I said sufficient care was not exercised by the parents when sending children to the hospital.

There have been no complaints from the parents. When I said there had been complaints it was from the house committee's report that cases which developed infection within a day or two after admission had been traced to coming from infected homes, and at the same time these parents signed a declaration that they had had no infection in their homes for six weeks, knowing perfectly well they had left a case of measles at home. I should thank you to publish the explanation.—Yours truly,

E. D. Spence.

Baildon, September 24, 1917.

[We willingly publish this statement. Though apparently parents have not yet complained, and indeed deserve censure for making false declarations, yet a hospital is certainly placed in difficulties which receives infected children, and will earn an unfortunate reputation in consequence. We suggested that the aid of the medical officer of health should be sought. A prejudice will in time be created against a hospital where infectious disease often breaks out, even in the minds of parents who are the real cause of the outbreaks. They will find it easier to blame the hospital than themselves. That is not justice, but it is human nature. Hence the urgent need of safeguarding the hospital's reputation in every way.—Ed. The Hospital.]

